# Impact of skin tone and cupping on erythema and thermal imaging measurements

**DOI:** 10.1038/s41598-025-24823-w

**Published:** 2025-11-27

**Authors:** Kathleen Jordan, Glory Tomi John, Andrew Chung, Miriam Asare-Baiden, Vicki Stover Hertzberg, Joyce C. Ho, Sharon Eve Sonenblum

**Affiliations:** 1 Nell Hodgson Woodruff School of Nursing, Emory University, Atlanta, GA USA; 2 Department of Computer Science, Emory University, Atlanta, GA USA

**Keywords:** Pressure injury, Erythema, Thermography, Thermal imaging, Cupping, Diseases, Health care, Medical research, Signs and symptoms

## Abstract

Erythema is commonly assessed to detect early signs of pressure injuries (PrIs). Thermography has emerged as a promising tool for PrI detection; however, correlations between temperature changes and erythema have not been consistent across skin tones. This study evaluated whether thermography and colorimetry could track temperature and erythema changes following cupping across diverse skin tones. Tissue changes were induced over a bony prominence using a cupping device at − 30 kPa for 5 min. Thermal images and colorimeter measurements were collected immediately after cupping and at one-minute intervals for seven minutes. Temperature at the cup rim increased 0.37 °C before returning to baseline while temperature dropped significantly at the cup center (− 0.54 °C), returning to baseline after 3–4 min. Erythema index increased immediately post-cupping (1.49 c.u.) and remained elevated throughout the seven-minute period. Neither temperature nor erythema changes varied by BMI or sex. Only erythema differed significantly across skin tone categories (*p* < 0.001). Regional variation in temperature responses suggests compressive and tensile stresses may generate distinct tissue effects with clinical relevance. Thermal imaging can identify subtle tissue changes not apparent during visual assessment, supporting its use as a complement to clinical evaluation for PrI prevention.

**Supplementary Information:**

The online version contains supplementary material available at 10.1038/s41598-025-24823-w.

## Introduction

 A Stage I pressure injury (PrI) is defined as area of non-blanching erythema of intact skin^1^. Erythema commonly presents as the reddening or discoloration of the skin due to a hyperemic response of tissue under pressure. It is often assessed during skin evaluations to detect early signs of a PrI. The presence of blanching erythema was found to have 75% sensitivity and 77% specificity at predicting the progression to a Stage I or higher PrI^2^. However, blanching may not be easily visible in darker skin tones, ^1^ which may lead to failure of early detection and delayed implementation of preventative techniques, causing longer hospital stays, infection, premature death^3^. It is also well documented that Blacks and African Americans are significantly more likely to develop PrIs, tend to develop them sooner after admission to a nursing home^4^, and are less likely to heal under the same treatment conditions^5^ compared to other racial and ethnic groups. Therefore, the use of various new bedside technologies has been recommended to improve early detection of PrIs regardless of skin tone^6,7^.

Once such technology under investigation is thermography or thermal imaging. Thermal imaging was found to be more effective at predicting PrI risk than the Braden scale^8^ and was able to identify previously undetected deep tissue injuries^9^. A decrease in sacral temperature by 0.1 degree compared to a healthy control region was associated with an increased risk of developing a PrI^8^. Peri-wound bed temperatures have also been correlated to wound healing trajectory^10^. Additionally, skin tone does not affect the thermal emissivity measured by thermal cameras^11^ and convolutional neural network models trained on thermal images after erythema induction detected temperature changes in individuals with darker skin tones more reliably than optical images^12^. Although thermal images were classified correctly more consistently than optical images, model performance varied across different skin tone groups^12^. While another study found no difference in temperature change after erythema induction across skin tones, a colorimeter detected significant differences in erythema measurements between skin tones^13^. To achieve a standardized erythema induction across diverse skin tones, both studies implemented cupping—a technique intended to facilitate consistent vascular responses and visible skin changes. Cupping is a traditional Chinese therapy that uses a negative pressure vacuum to pull a small area of skin into a cup. Cupping treatment increases blood volume and tissue oxygenation to the tissue under the cup while slightly decreasing the blood volume and oxygenation in the surrounding tissue^14^. The blood flow changes also lead to changes in skin temperature under the cupping region, with studies reporting an initial decrease of 0.4 °C followed by increases of 0.4–1.42 °C above the pre-cupping baseline temperature^15^. Additionally, cupping can cause changes in erythema and skin color in the region under pressure, although the effects do not necessarily align with the observed temperature changes. Under laboratory conditions, −25 to −30 kPa of pressure produced a significant increase in erythema at the forearm and ulnar head immediately after cupping, which was sustained above baseline after 5 min of recovery. However, the effects differed slightly by skin tone group^13^.

The disconnect between measured changes in erythema and skin temperature only scratches the surface of the complicated hemodynamics initiated by cupping and other pressure-induced microvascular tissue damage. The observed mismatches between temperature and erythema responses across different studies suggest our understanding of these relationships is incomplete. Many studies examining the hemodynamic effects of cupping used only male participants^14^ and did not report skin tone, race, or ethnicity, ^14–16^ despite the difficulty of visualizing erythema in darker skin tones. Additionally, many studies have focused on single, discrete timepoints immediately or very shortly after the removal of the cupping pressure^13^, leaving the temporal patterns of tissue recovery largely unexplored. Furthermore, while individual studies have examined either temperature or erythema responses directly under the cupping region, comprehensive spatial and temporal characterization of both measures across diverse skin tones has not been investigated. To address these knowledge gaps, this study aimed to characterize the spatial and temporal patterns of temperature and erythema responses following controlled cupping across individuals with diverse skin tones. Specifically, the objectives were to:

1. Quantify the changes in temperature and erythema over an extended recovery period,

2. Investigate the mismatch between temperature and erythema responses observed in prior studies, and.

3. Evaluate how temperature and erythema responses to low pressure cupping vary across different skin tone groups.

## Results

### Participants

This study included a total of 35 healthy adult volunteers, aged 18 to 73 (Table 1). To ensure a variety of skin tones, participants were recruited into two groups based on their Monk Skin Tone (MST) Scale level when measured at the inner forearm^17^. Thirty participants were included in the darker skin tone group (MST level 6 or higher) and 5 were included in the lighter skin tone group (MST level 5 or lower). Skin tone at the inner forearm was also quantified using the Melanin Index measured by the SkinColorCatch^®^ (Delfin Technologies Ltd, Kuopio, Finland) and then classified using the modified Eumelanin Human Skin Colour Scale (Eumelanin Scale-Modified)^18^ which ranges from Eumelanin Low (Melanin Index < 25) to Eumelanin High (Melanin Index ≥ 100).

**Table 1:** Participant characteristics.

Characteristic	N = 35^1^
**Age (Years)**	39.71 ± 16.45
Sex	
Female	24 (69%)
Male	11 (31%)
**BMI (kg/m** ^**2**^ **)**	29.41 ± 6.83
BMI Category	
Normal	13 (37%)
Overweight	8 (23%)
Obese	14 (40%)
Race	
American Indian or Alaskan Native	2 (5.7%)
Asian	5 (14%)
Black or African American	26 (74%)
White	6 (17%)
More than one race	3 (8.6%)
Ethnicity	
Hispanic or Latino	2 (5.7%)
Not Hispanic or Latino	33 (94%)
Smoking Status	
Former smoker	6 (17%)
Has never smoked	29 (83%)
Modified Eumelanin Skin Tone Category	
Intermediate Low	4 (11%)
Intermediate	6 (17%)
Intermediate Mid	22 (63%)
Intermediate High	3 (8.6%)
**Melanin Index**	55.94 ± 13.54
Monk Skin Tone Group	
2	3 (8.6%)
4	1 (2.9%)
5	1 (2.9%)
6	7 (20%)
7	22 (63%)
8	1 (2.9%)

### Temperature and erythema after cupping

**Fig. 1:**
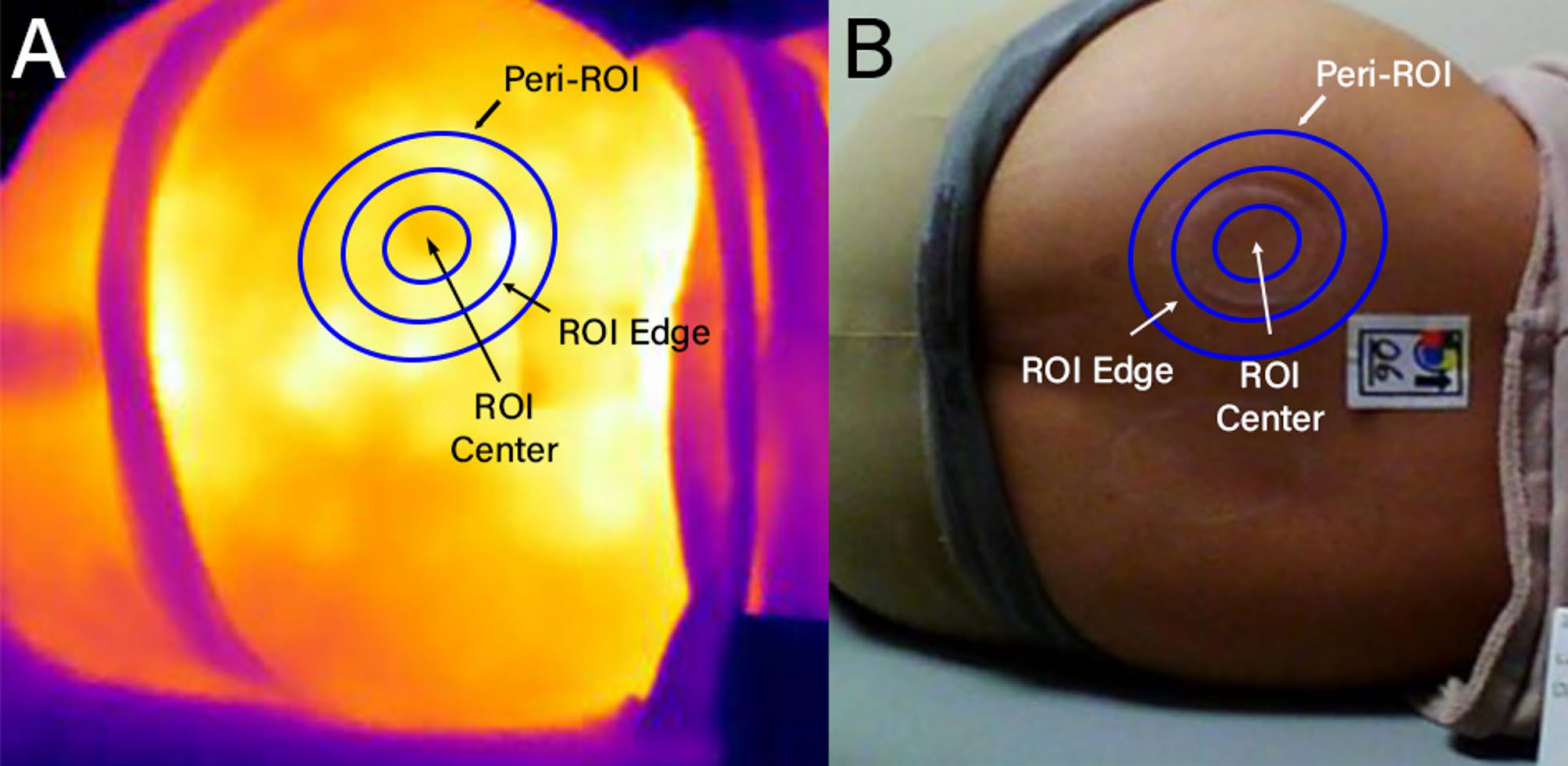
The regions of interest identified for the cupping protocol are illustrated on the (**A**) thermal image and (**B**) optical image following alignment via transformation. Regions were concentric ellipses.

Tissue changes over the posterior superior iliac spines (PSIS) were induced by using a 2” diameter cupping device and maintaining a −30 kPa pressure for 5 min. After this, thermal images and colorimeter measurements were taken starting immediately after the removal of cupping device (0 min post-cupping) and then every minute for 7 min. To process the cupping data, 3 concentric regions of interest (ROIs) were identified (Fig. 1) to study how temperature changed within and around the ROI. The ROI Center contained the region within the smallest central ellipse and represents the tissue under most deformation during cupping. The ROI Edge includes the region between the ROI Center and the outer edge of the tissue under the cup and represents the tissue under the rim of the cup during cupping. The Peri-ROI includes the region between the outermost ellipse and the ROI Edge (i.e. the region under the rim of the cup) and represents the adjacent healthy tissue not under pressure during cupping.

**Fig. 2:**
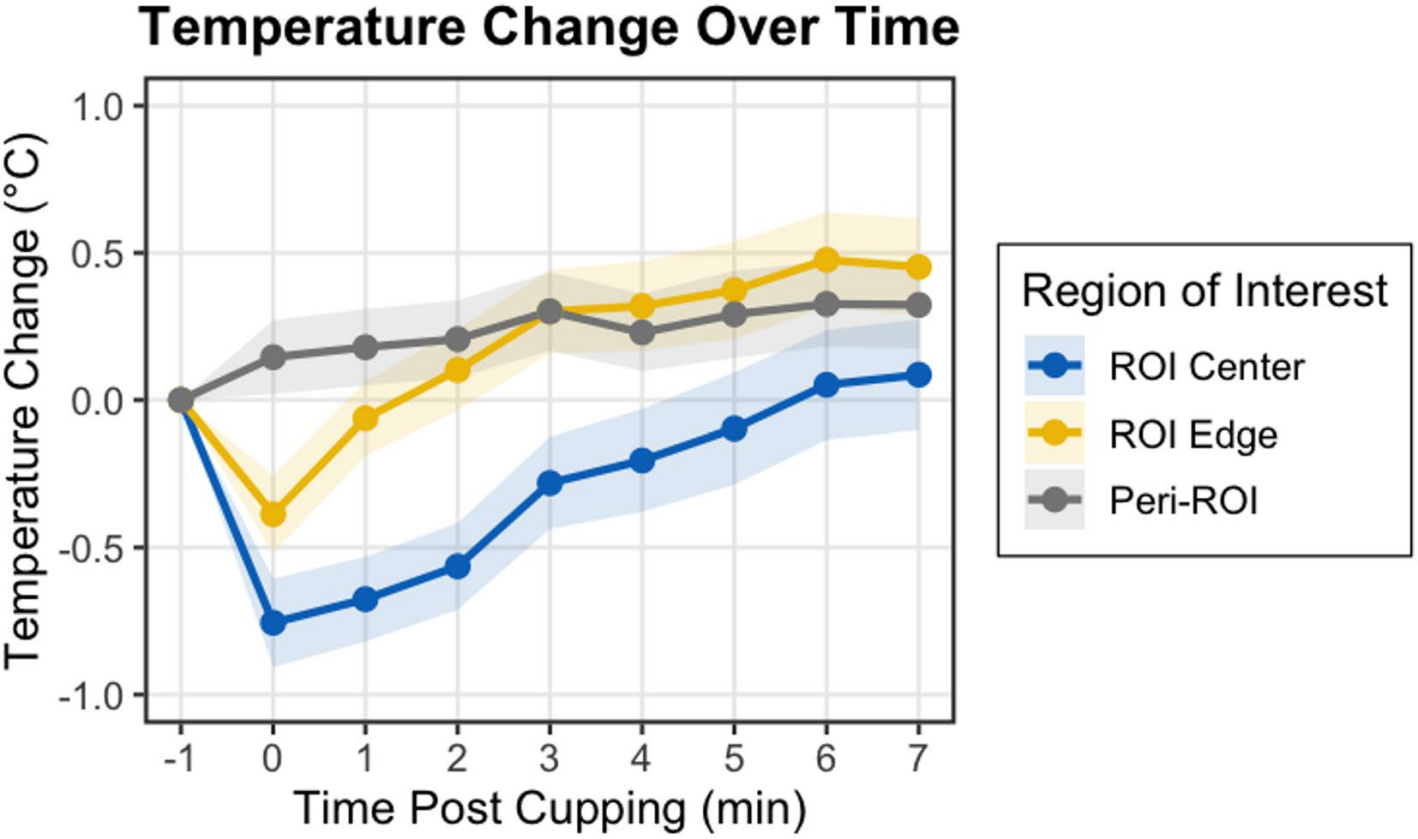
Temperature changes relative to pre-cupping baseline in concentric regions of interest before and after cupping.

Immediately following cupping, the median temperature in the Peri-ROI increased by 0.37 °C (interquartile range: −0.17 °C to 0.61 °C) relative to baseline (Fig. 2). In contrast, the temperature in the tissue beneath the rim of the cup (ROI Edge) and in a small central region within the cup (ROI Center) decreased by − 0.06 °C (IQR: −0.46 °C to 0.26 °C) and − 0.54 °C (IQR: −1.01 °C to − 0.09 °C), respectively. At 0 min post-cupping, neither the temperature in the Peri-ROI nor the ROI Edge changed significantly from baseline, but the temperature in the ROI Center significantly decreased (one-sample t-test, *p* < 0.05). As shown in Fig. 2, the temperature in the Peri-ROI returned to baseline within a minute and remained there for the duration of testing. The ROI Edge increased back to baseline by 1 min and continued to increase above baseline temperature for the duration of testing. The smaller ROI Center took longer to return to baseline temperature (approximately 3–4 min) and continued increasing past baseline for the duration of testing.

Unlike temperature, the erythema index (measured at a single point at the center of the ROI) increased by 1.49 c.u. from baseline immediately after cupping and stayed consistently above baseline for the entire 7 minutes of testing (Table 2) (*p* < 0.001). There was a weak correlation (Pearson corr = 0.338, *p* < 0.001, 95% CI [0.221, 0.445]) observed between the change in erythema and the change in temperature. Statistical analysis was not run on the erythema index over time by skin tone group due to limited sample size in the lighter skin tone group.

**Table 2:** Change in erythema index from baseline after cupping. Across all subjects, the erythema index was significantly increased from baseline at all timepoints post-cupping (*, *p* < 0.001).

	Minutes from Cupping							
**Subjects**	**0**	**1**	**2**	**3**	**4**	**5**	**6**	**7**
All subjects (*N* = 35)	1.49 ± 2.04^*^	1.37 ± 2.14^*^	1.63 ± 2.09^*^	1.56 ± 1.94^*^	1.56 ± 1.85^*^	1.65 ± 1.84^*^	1.57 ± 1.84^*^	1.39 ± 2.01^*^
MST 6–10 (*N* = 30)	1.11 ± 1.76	0.98 ± 1.90	0.68 ± 3.53	1.17 ± 1.70	1.21 ± 1.61	1.29 ± 1.55	1.20 ± 1.54	1.02 ± 1.75
MST 1–5 (*N* = 5)	3.80 ± 2.23	3.69 ± 2.19	3.99 ± 1.89	3.88 ± 1.78	3.67 ± 1.94	3.80 ± 2.20	3.80 ± 2.13	3.59 ± 2.27

**Fig. 3:**
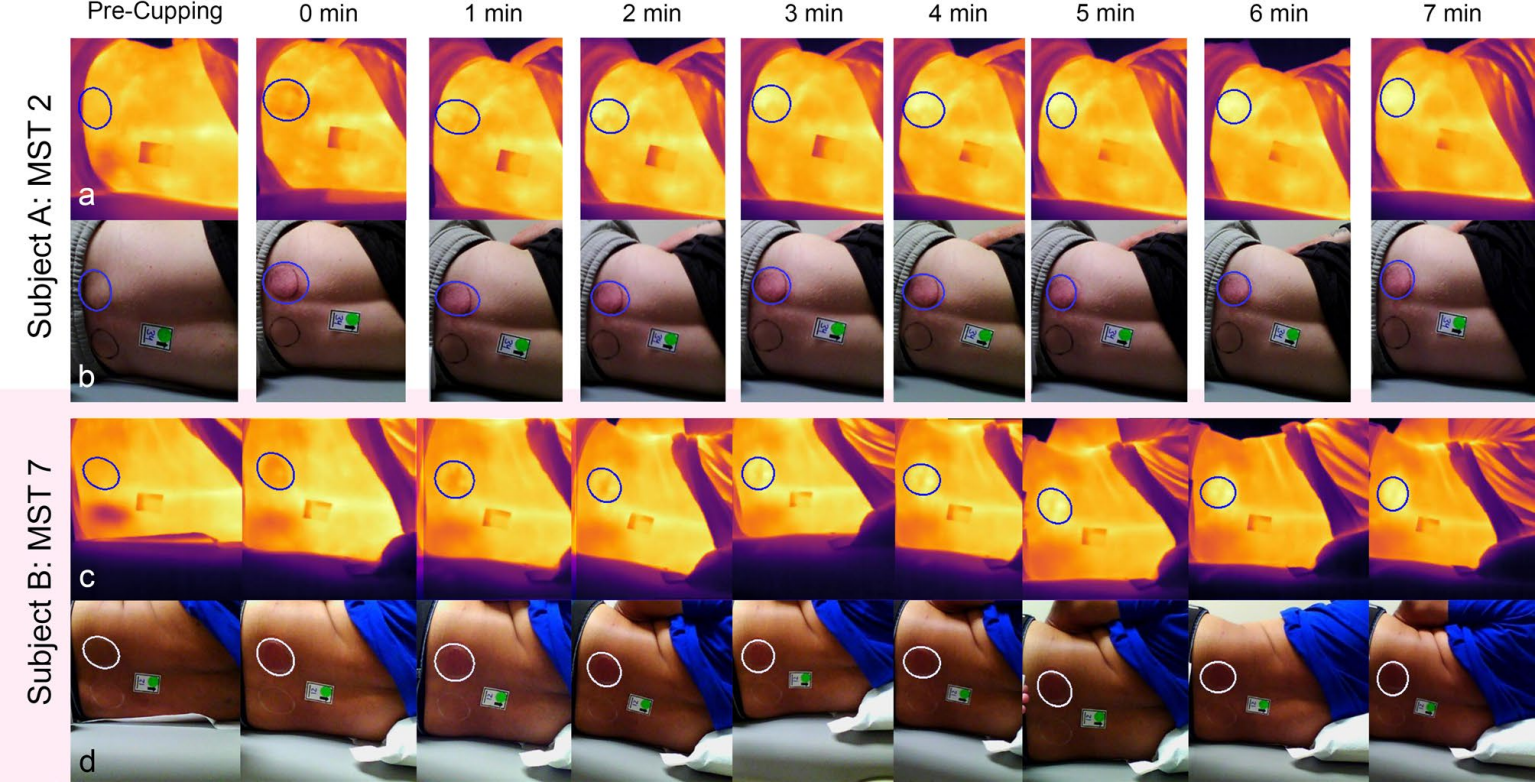
Thermal (**a** & **c**) and optical (**b** & **d**) representation of cupping over time, with the ROI Edge marked, demonstrates the temperature and erythema changes in a participant with light (Subject A, a & b) and dark (Subject B, c & d) skin tones. Subject B’s optical images were uniformly brightened for demonstration purposes only; these adjustments were not used in any analysis.

Figure 3 illustrates the visual appearance of erythema changes in two representative subjects: Subject A (lighter skin tone group) shows clear visible erythema, while Subject B (darker skin tone group) demonstrates the difficulty of visually detecting erythema despite measurable increases. Subject B averaged an increase of 3.43 ± 0.46 c.u. from baseline across the 7-minute recovery period while Subject A averaged an increase of 5.92 ± 0.39 c.u.

### Temperature and erythema after cupping – differences across subjects

**Fig. 4:**
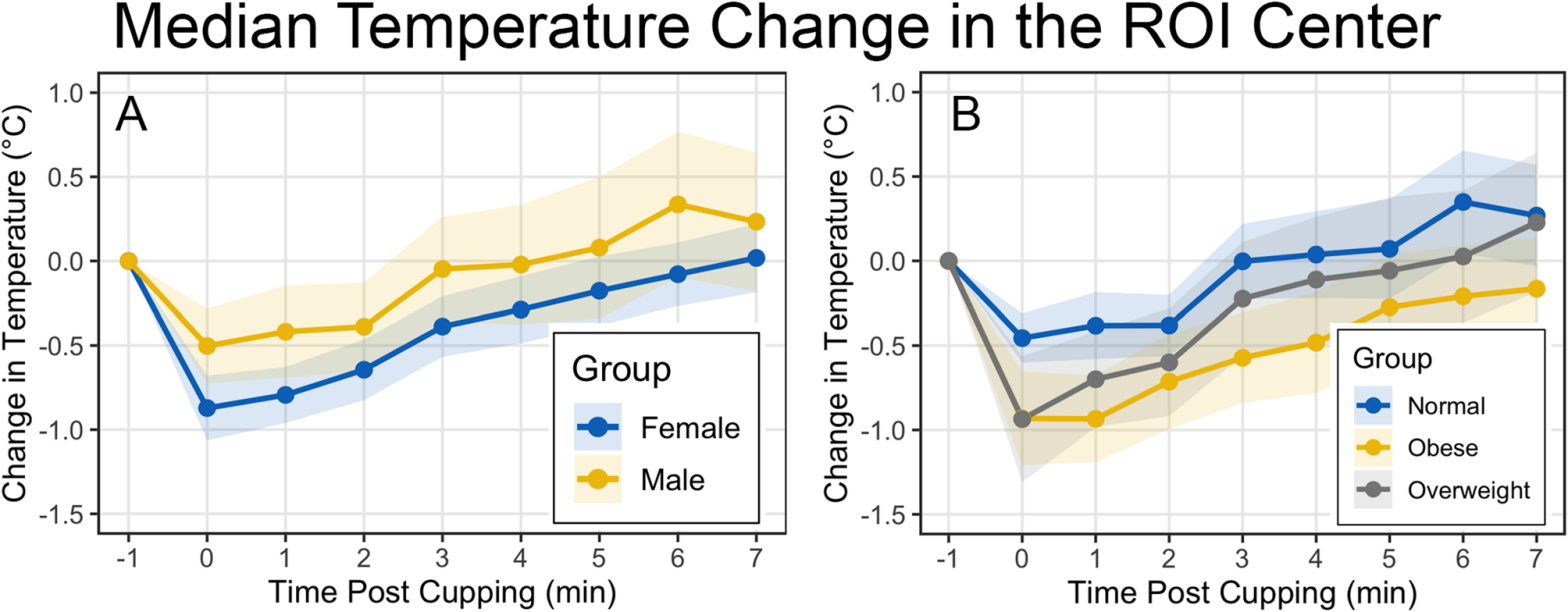
Sex (**a**) and BMI category (**b**) were not associated with significant differences in temperature change following cupping, but trends immediately following cupping were evident.

**Fig. 5:**
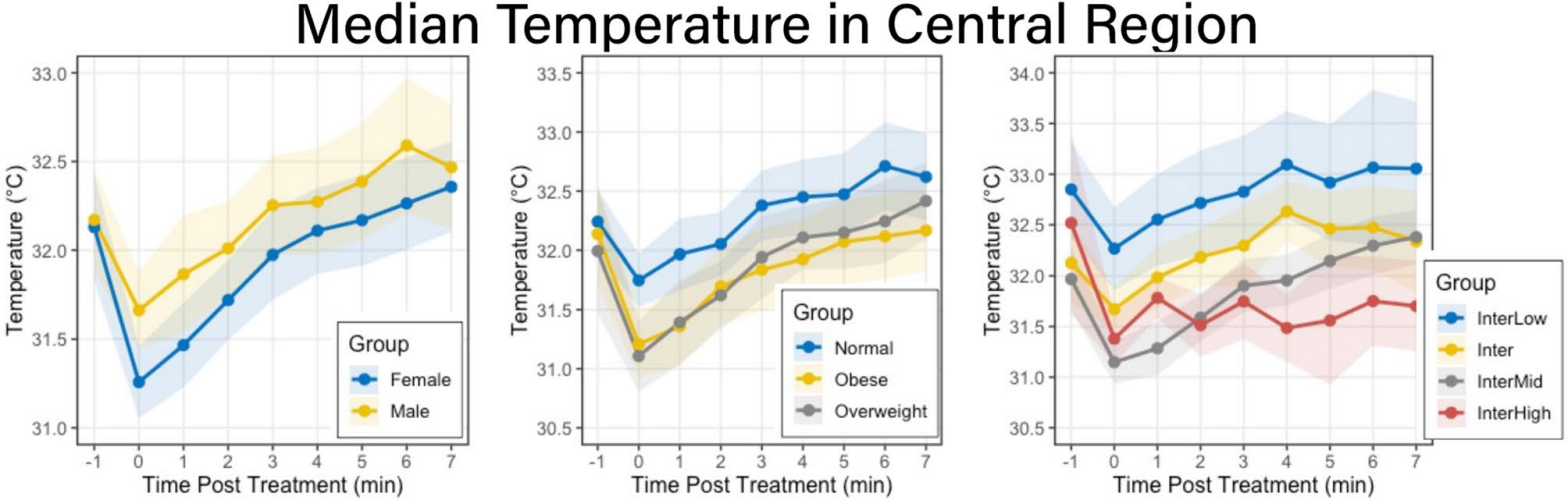
Modified Eumelanin Skin Tone Category was not associated with differences in temperature change following cupping, despite apparent trends visible in the data (**a**), but erythema response was significantly different across skin tone (**b**). The weak correlation between erythema and temperature change was most pronounced in the Intermediate Low and Intermediate skin tone categories (**c**). Legend: InterLow = Intermediate Low, Inter = Intermediate, InterMid = Intermediate Mid, InterHigh = Intermediate High.

Because the largest reduction in temperature was noted at 0 min in the small ROI Center, subject characteristics were studied in this region across all timepoints. No significant differences in temperature were observed across sex, BMI category, or skin tone at any time point, despite the trends visible in Figs. 4 and 5a at 0 min post-cupping or as seen visually in Fig. 3.

Measurements of change in erythema did not vary across sex or BMI category (not shown) but did vary significantly across skin tone category (*p* < 0.001, Fig. 5b). The correlation between change in erythema and change in temperature from baseline, while always weak, was dominated by participants in the Intermediate Low and Intermediate skin tone categories, the two lightest skin tone groups in this study (Fig. 5c). Participants in the Intermediate Mid group did not demonstrate this relationship. While there were only 3 participants in the Intermediate High group, the darkest skin tone group in this study, amongst their 21 data points, there was a trend towards a negative correlation between change in temperature and change in erythema (Pearson corr = −0.421, *p* = 0.057, 95% CI [−0.721, 0.013]).

## Discussion

This study characterized the spatial and temporal patterns of temperature and erythema responses following low-pressure cupping across individuals with diverse skin tones. Literature indicates that cupping causes a decrease in skin temperature during suction measured at a bony prominence^13^ or with adequate pressure ^16,19^. The maximum increase in temperature following cupping has been reported from 0.35 °C to 1.42 °C ^13,19^, varying by body site and cupping pressure. Similarly, in this study, temperature within the ROI Edge, (which represents the area under the rim and directly inside of the cup) increased over time, presumably as blood flowed to the region in a reactive hyperemic response^20^. Temperature surrounding the cupping region (Peri-ROI) experienced little temperature deviation at any time, while the ROI Center dropped significantly right after cupping and took 3–4 min to return to baseline.

One possible explanation for the differences in temperature responses across the studied regions is the heterogeneity of the mechanical strain applied to the tissue during cupping. The stress in the ROI Edge region is the highest in magnitude but is compressive^21^. This suggests that tissue compression temporarily occludes the capillaries without causing damage or rupture, leading to an initial drop in temperature from ischemia that rapidly returns to baseline once the pressure is released and blood flow resumes. The ROI Center, however, is under high tensile stress, which is believed to lead to greater accumulation of blood and possible rupture of capillaries^21^. The large cup diameter leads to greater skin displacement within the cup, pulling on progressively deeper layers of the tissue and distributing the stress more extensively throughout the skin^21,22^. The extended recovery time seen in this region is likely due to the increased vascular damage caused by mechanical stress and strain affecting deeper levels of tissue, resulting in longer repair times, slower effective reperfusion to the area, and potentially leading to an acute inflammatory response. The spatial and temporal changes in temperature documented in this study offers insight into the hemodynamics of healthy tissue healing after temporary, pressure-induced microvascular damage.

These distinct physiological responses observed during cupping have important implications for understanding what thermal imaging actually measures in clinical PrI assessment. Thermal imaging has been used for early detection of PrIs, where either localized increases or decreases in relative temperature may indicate signs of early damage, and is recommended as a supplement to visual skin assessments with the same goal^1,23^. However, our results demonstrated that temperature and erythema changes following cupping were only weakly correlated, suggesting that these responses may be driven by different physiological mechanisms or occur at different stages of tissue damage or healing. The rupture of capillaries under the tensile stress and strain within the cup is believed to be the main cause of visible erythema, as blood accumulates within the damaged tissue ^22^. In contrast, the skin under the compressive force of the rim of the cup does not develop erythema, as the capillaries are not ruptured^21^, but does experience temperature change, likely ischemia induced by the compression at the cup’s rim ^16^. Removing the pressure is enough to restore blood flow to the undamaged capillaries in this region and trigger a rise in temperature; however, it does not immediately clear the excess blood responsible for visible erythema at the center region. In clinical use, thermal imaging may be best to detect ischemic areas immediately after pressure is removed, providing prompt feedback about which regions may be at risk. However, these thermal changes may be transient in cases of reversible ischemia, while persistent thermal abnormalities could indicate more significant tissue damage. In contrast, non-blanchable erythema persists much longer after the pressure is relieved, making it more difficult to pinpoint the exact cause or timing of the injury, but it offers visual confirmation that some degree of chronic tissue damage has occurred, and protective interventions need to be implemented. Therefore, thermal imaging and visual assessment may be complementary rather than redundant tools for clinical assessment. Thermal imaging may be used for the early detection of ischemic tissue even before permanent damage has occurred, providing the opportunity to intervene and off load pressure before the vasculature is fully compromised and erythema develops. On the other hand, visual and thermal assessment together may better track the progression or healing of damage that has already occurred, especially when visual changes alone are not readily apparent or reliable.

Thermal imaging’s ability to provide valuable additional information about underlying physiological processes independent of skin tone makes it an important tool for early detection for all patients, but especially for individuals with darker skin tones. Previous work showed that the erythema index increased following cupping^13^, and our current study confirmed this pattern in participants with most skin tones. However, participants with the darkest skin tones showed a decrease in erythema index after negative pressure application. This supports the results of another study that found that capillary refill or blanching of erythema alone was insufficient to determine progression of intact skin to necrosis^9^. Rather than melanin simply masking the visible effects of erythema in darker skin tones, these different responses may reflect genuine differences in underlying physiological processes. This inverse erythematous response in the darkest skin tone group suggests that participants with the darkest skin tones may have experienced different changes in hemoglobin levels, the physiological component measured by the colorimeter to quantify erythema, caused by changes in blood flow as compared to those usually documented after cupping^14^. Nevertheless, the observed temperature changes clearly indicated physiological responses to cupping, consistent with previous research on localized changes in microcirculation and oxy- and deoxy-hemoglobin^14,20^. These findings demonstrate that thermal imaging detects underlying tissue changes that visual assessment cannot capture across diverse skin tones, supporting its use as a complement to, rather than replacement for, visual assessment.

Study limitations include the small population size, particularly for the darkest skin tone group, inclusion of only healthy adults, the use of cupping rather than actual PrI conditions, and the limited seven-minute recovery period. Future research should examine larger populations, including individuals at risk for PrI development, with different tissue characteristics, and compare clinical loading conditions over extended timeframes.

## Conclusion

This study successfully characterized the spatial and temporal patterns of both temperature and erythema responses across diverse skin tones, quantified their changes over a seven-minute recovery period, and reinforced the previous findings that temperature and erythema are not always correlated, particularly in people with darker skin tones. These findings have important clinical implications. The observation that tensile stress and strain from cupping may produce longer-lasting effects than compressive stress is clinically significant, since current prevention strategies often target reducing compression while overlooking the shear and tensile stresses at the interface and resultant internal shear strains from patient positioning^24^. This work highlights the importance of expanding our understanding of how temperature and erythema relate to different physiological processes, providing a foundation for interpreting thermal imaging’s clinical implications in patients at risk for PrI development.

## Methods

This study used a pre-post experimental design that induced erythema via cupping on the lower backs of healthy adults. Informed consent was obtained from all participants. All methods were conducted in accordance with relevant guidelines and regulations. All protocols received ethical approval from the Emory University Institutional Review Board (eIRB number 00005999). All data were anonymized with assigned identification codes. Participants were informed that their participation was entirely voluntary and that they could withdraw from the study at any point without providing any justification.

### Equipment and measurements

#### Demographic data

Participants completed an electronic REDCap survey containing questions about demographic data and information about height, weight, and smoking status.

#### Colorimetry

The SkinColorCatch^®^ (Delfin Technologies Ltd, Kuopio, Finland), a digital colorimeter, was used to measure the Melanin Index of the participant’s forearm, and to describe the Erythema Index of their posterior superior iliac spine (PSIS) throughout testing. Skin tone at the inner forearm were converted to the ColorMeter DSM II Melanin Index as described in a previous publication^25^ and classified using the Eumelanin Scale-Modified categories^13,18^, adapted from the scale initially described by Dadzie, et al. ^26^.

#### Thermal imaging

The FLIR E8-XT (FLIR Systems, Inc., Wilsonville, OR) thermal camera was used to collect optical and thermal images of the sacral region throughout the study.

### Data collection

The participants’ PSIS were palpated while the participant was standing. A 2” circle was drawn on the skin around each PSIS using a pencil with high color contrast to the participant’s skin tone to ensure a solid and clear marking of the ROI. The left PSIS was used for the cupping protocol. A sticker with the subject ID was placed on the participant at midline superior to the sacral region.

**Fig. 6:**
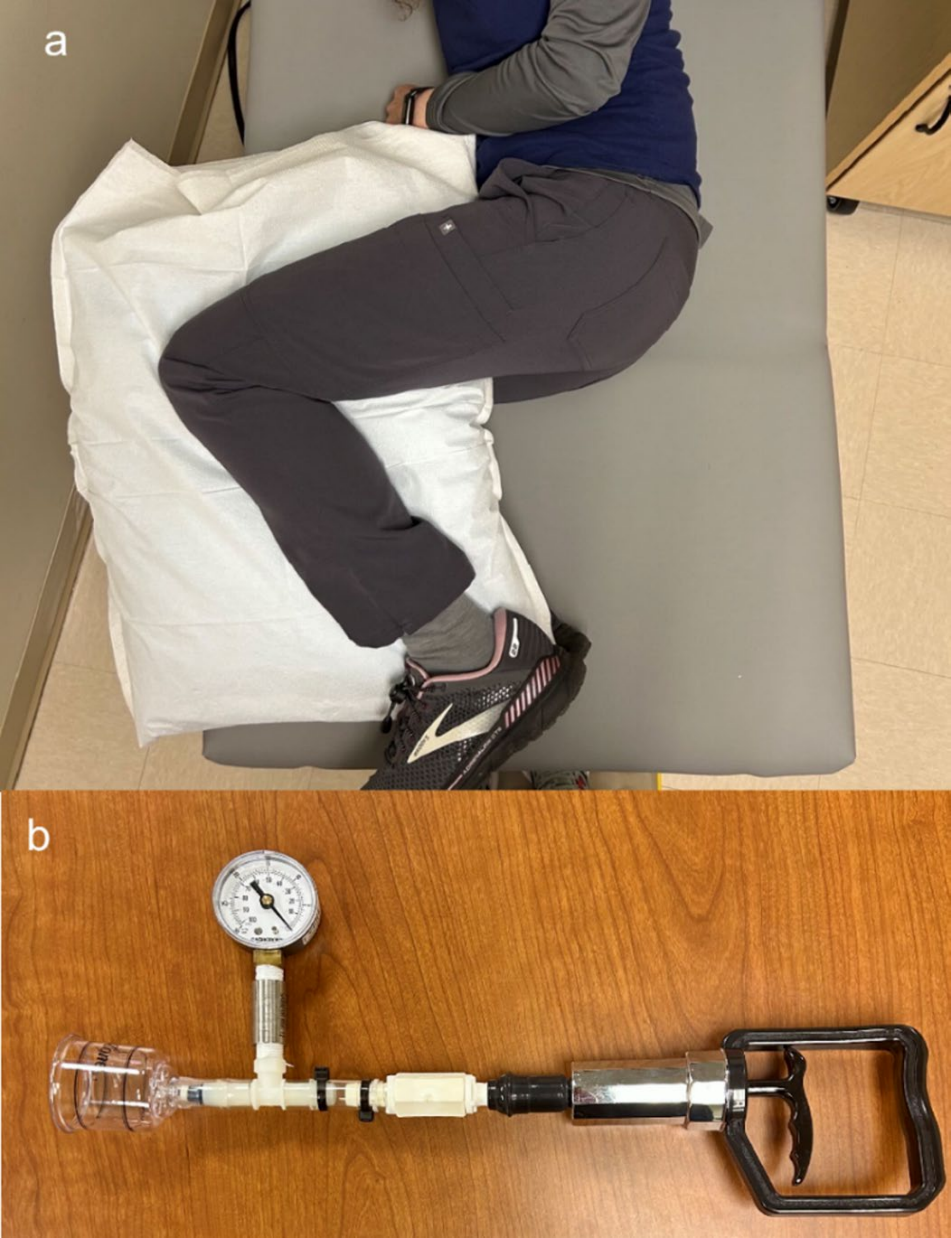
Study setup and equipment. (**a**) Participant position during image collection. (**b**) Device used to maintain consistent pressure application during cupping with a 2” diameter cup connected to a manometer to monitor pressure levels, and a hand pump used to create the negative pressure vacuum.

A single baseline image (pre-cupping) was taken with the FLIR E8-XT thermal camera at 50 cm, with ambient lighting, and with the participant in a side-lying position with their hips and knees bent to approximately 90 degrees, legs on top of one another and a pillow placed between their knees to simulate a clinically relevant and repeatable posture (Fig. 6a). The SkinColorCatch^®^ was used to collect a single baseline erythema value at the center of the PSIS ROI. Participants then rotated back to prone where the cupping procedure was conducted on their left PSIS. Tissue changes were induced within the ROI by using a 2” diameter cupping device and maintaining a −30 kPa pressure for 5 min (Fig. 6b). After this, participants returned to the knees stacked posture and thermal images were collected under the same conditions starting immediately (0 min post-cupping) and then every minute for 7 min. The SkinColorCatch^®^ was also used at the same intervals to measure the Erythema Index at the center of the ROI. This completed the cupping protocol for a total of 9 images and 9 erythema measurements, 1 baseline and 8 post-cupping.

### Data processing

The corner points on the identification card and/or sticker visible in the images were mapped between the optical and thermal images with an affine transformation, aligning the two images. Three concentric ROIs were identified (Fig. 1) on the images to study how temperature changed within and around the ROI. An elliptical region of interest was selected using a custom Python script following the outer edge of the imprint left by the cup. Two more ellipses were defined based on the dimensions of the ROI drawn on the image – one with axes 1.5x the original and one with axes 0.5x the original (approximately 0.25x area) – and all shared a common center point. The Peri-ROI included the region between the outermost ellipse and the indent left by the rim of the cup. The ROI Edge included the region between indent left by the rim of the cup and smallest ellipse. The ROI Center contained the region within the smallest ellipse.

### Data analysis

Temperature and erythema changes were calculated by subtracting baseline values (pre-cupping) from measurements taken at 0–7 min post-cupping. One-sample t-tests (m = 0) were conducted to determine if temperature and erythema changes were significantly different from zero. The relationship between erythema and temperature was assessed using Pearson correlation, including only measurements from 1 to 7 min post-treatment. Analysis of variance (ANOVA) was used to examine differences in temperature changes at 0 and 7 min across participant characteristics including sex, BMI category, and Modified Eumelanin Skin Tone Category. The relationship between erythema and thermography measurements was evaluated using Pearson correlation and visualized according to skin tone category.

## Electronic Supplementary Material

Below is the link to the electronic supplementary material.

Supplementary Information

## Data Availability

The summary statistics generated during and/or analyzed during the current study is available on Zenodo (https://doi.org/10.5281/zenodo.17399275) .
