# Correction: Impact of skin tone and cupping on erythema and thermal imaging measurements

**DOI:** 10.1038/s41598-026-65527-z

**Published:** 2026-08-06

**Authors:** Kathleen Jordan, Glory Tomi John, Andrew Chung, Miriam Asare-Baiden, Vicki Stover Hertzberg, Joyce C. Ho, Sharon Eve Sonenblum

**Affiliations:** 1 Nell Hodgson Woodruff School of Nursing, Emory University, Atlanta, GA USA; 2 Department of Computer Science, Emory University, Atlanta, GA USA

Correction to: *Scientific Reports* 10.1038/s41598-025-24823-w, published online 27 November 2025

The original version of this Article contained an error in the description of the concentric regions of interest in the Methods section, under the subheading ‘Data processing’. Consequently,

“Two more ellipses were defined based on the dimensions of the ROI drawn on the image – one with axes 1.25x the original and one with axes 0.25x the original – and all shared a common center point.”

now reads:

“Two more ellipses were defined based on the dimensions of the ROI drawn on the image – one with axes 1.5x the original and one with axes 0.5x the original (approximately 0.25x area) – and all shared a common center point.”

In addition, the cupping regions in Figure 1 have been updated. Figures 2, 4 and 5 have been regenerated using the originally intended ROI dimensions (25%/125% axes). The original Figures and accompanying legends appear below.

**Fig. 1:**
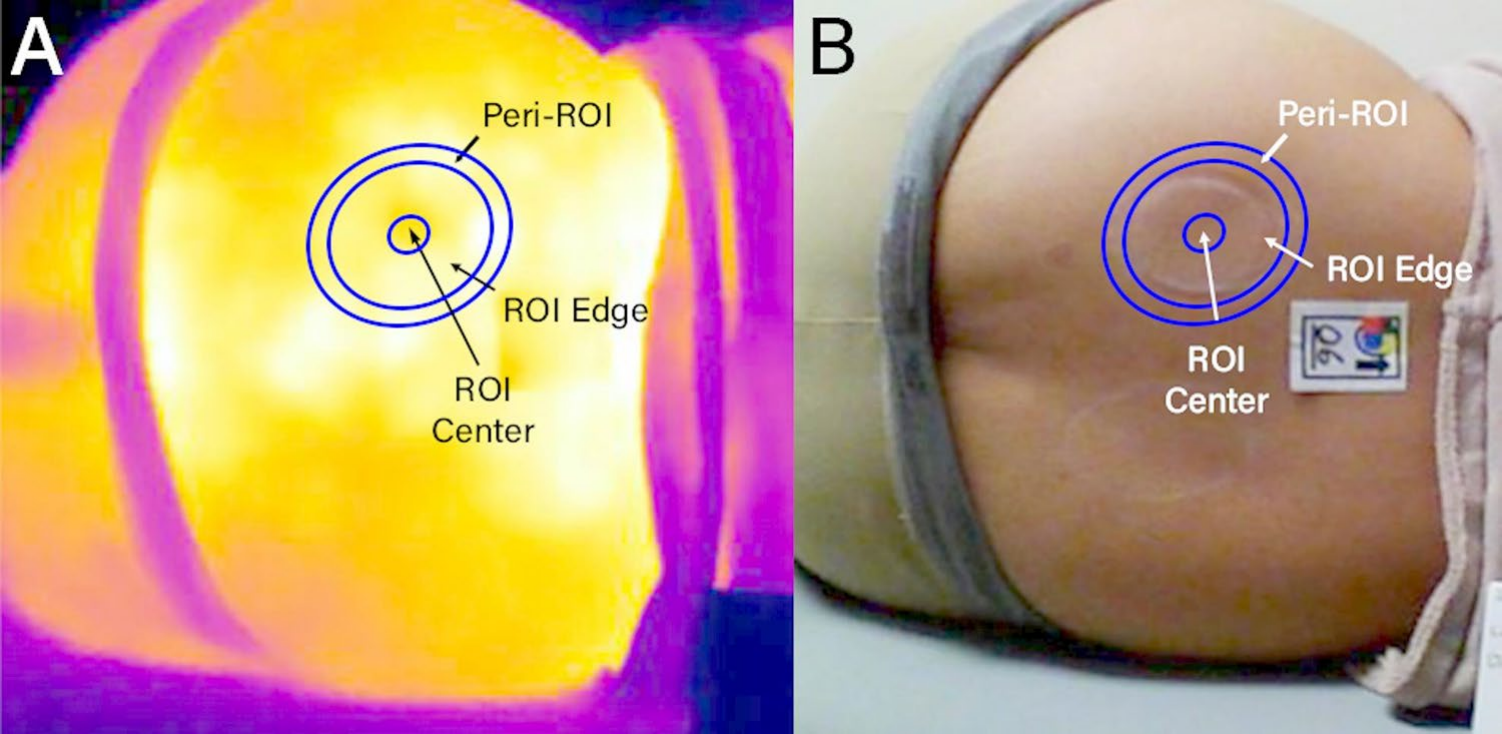
The regions of interest identified for the cupping protocol are illustrated on the (**A**) thermal image and (**B**) optical image following alignment via transformation. Regions were concentric ellipses.

**Fig. 2:**
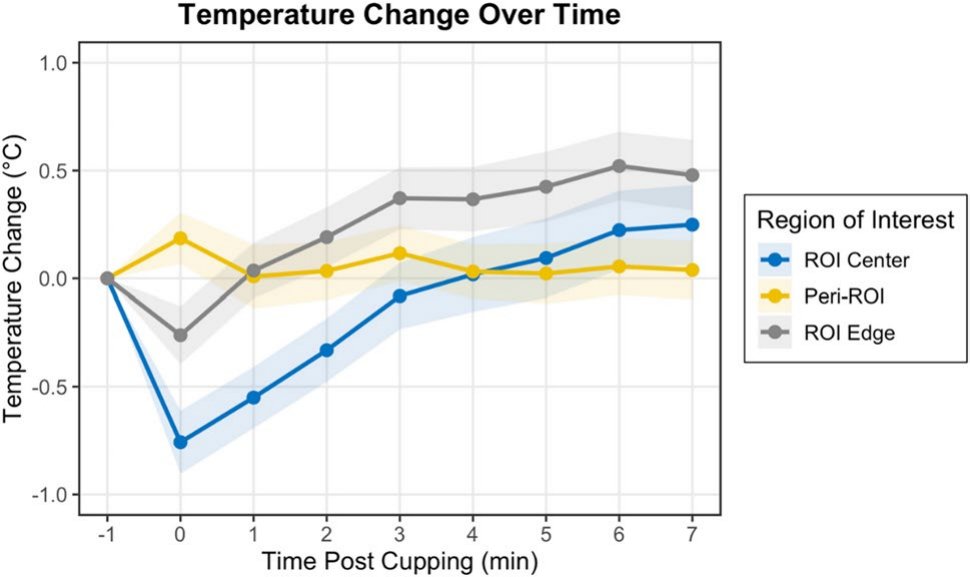
Temperature changes relative to pre-cupping baseline in concentric regions of interest before and after cupping.

**Fig. 4:**
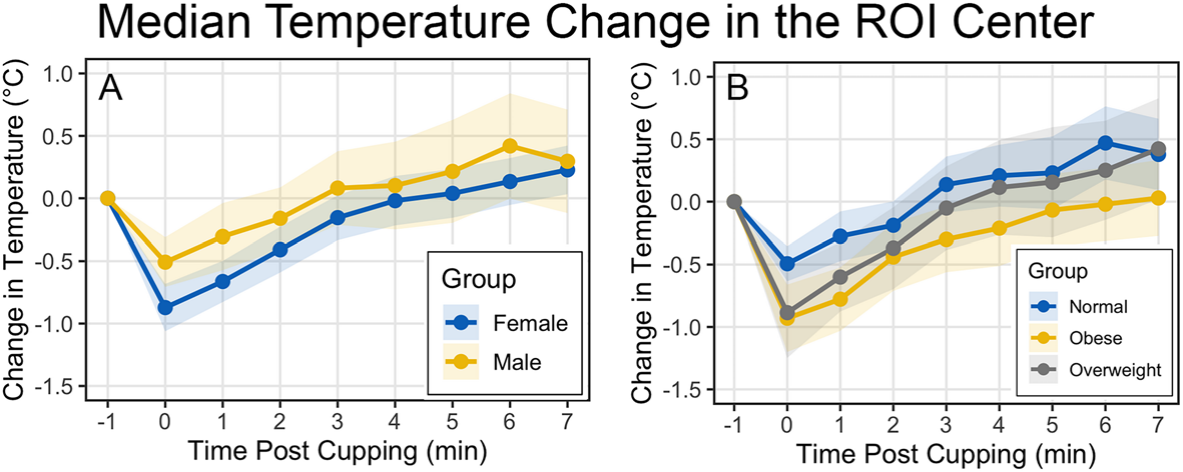
Sex (**a**) and BMI category (**b**) were not associated with significant differences in temperature change following cupping, but trends immediately following cupping were evident.

**Fig. 5:**
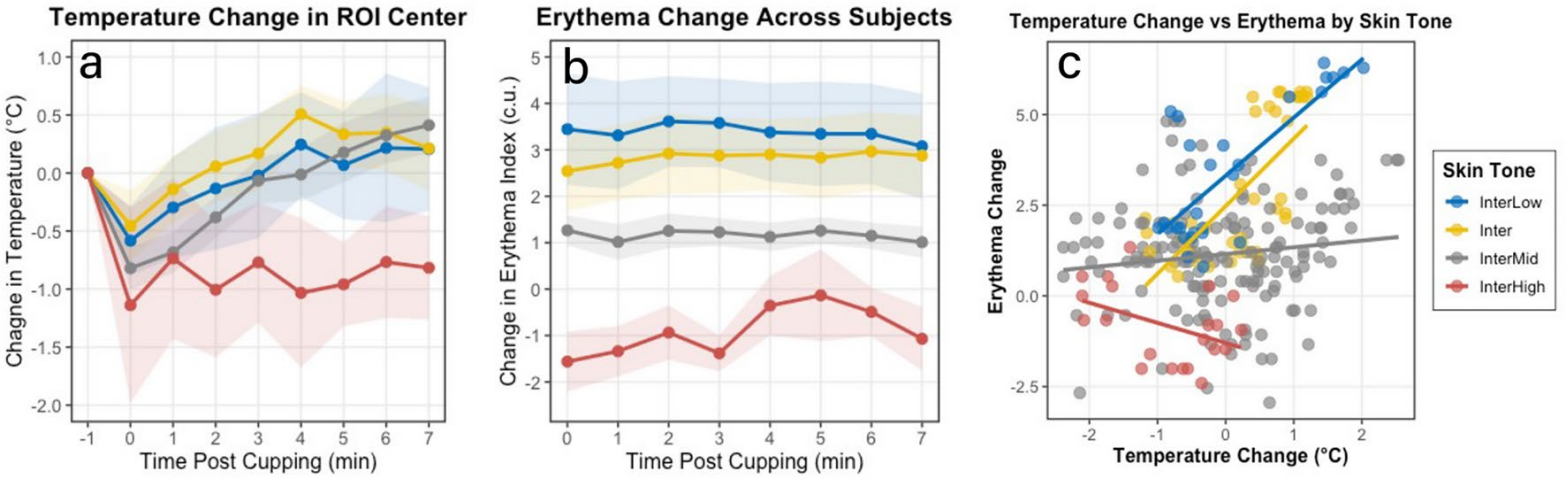
Modified Eumelanin Skin Tone Category was not associated with differences in temperature change following cupping, despite apparent trends visible in the data (**a**), but erythema response was significantly different across skin tone (**b**). The weak correlation between erythema and temperature change was most pronounced in the Intermediate Low and Intermediate skin tone categories (**c**). Legend: InterLow = Intermediate Low, Inter = Intermediate, InterMid = Intermediate Mid, InterHigh = Intermediate High.

The authors also reanalyzed the data using both ROI definitions (published dimensions and originally intended 25%/125% axes). A comparison of key values is provided in the Supplementary Information file.

The original Article has been corrected.

