# Supplementary material for: Impact of skin tone and cupping on erythema and thermal imaging measurements
Source: Sci Rep. 2025 Nov 27;15:42575. doi: 10.1038/s41598-025-24823-w (PMC12663286; doi:10.1038/s41598-025-24823-w)
Supplement: Supplementary file 1 [file 41598_2025_24823_MOESM1_ESM.docx]

**Supplement S1.**

**Comparison of Results Using Two ROI Definitions**

Values are presented for the published analysis (ROI Center = 50% Outer ROI Edge Axes, Peri-ROI = 150% Outer ROI Edge Axes) and verification analysis (ROI Center = 25% Outer ROI Edge Axes, Peri-ROI = 125% Outer ROI Edge Axes). Both methods yield consistent findings with identical conclusions.

**1. Temperature Changes from Baseline at 0 Minutes Post-Cupping**

| **Region** | **Published (25% Area)** | **Corrected (25% Axes)** | **Change** |
| --- | --- | --- | --- |
| Peri-ROI | 0.37°C (IQR: −0.17 to 0.61°C) | 0.15°C (IQR: −0.12 to 0.67°C) | ↓ 0.22°C |
| ROI Edge | −0.06°C (IQR: −0.46 to 0.26°C) | −0.39°C (IQR: −0.59 to 0.16°C)* | ↓ 0.33°C |
| ROI Center | −0.54°C (IQR: −1.01 to −0.09°C)* | −0.76°C (IQR: −1.26 to −0.26°C)* | ↓ 0.22°C |

**p < 0.01 vs. 0 (one-sample t-test). Note: ROI Edge is now statistically significant in the verification analysis.*

Figure 2. Temperature changes relative to pre-cupping baseline in concentric regions of interest before and after cupping. (Revised to use intended 125%/25% axes)

**2. Temperature Recovery Trajectories**

| **Region** | **Published Finding** | **Corrected Finding** | **Interpretation** |
| --- | --- | --- | --- |
| ROI Center | Returned to baseline at ~3–4 min | Returned to baseline at ~6 min | ~2-min delay in recovery |
| ROI Edge | Returned to baseline by 1 min, then increased above baseline | Nearly returned to baseline by 1 min, then increased above baseline | Consistent pattern |
| Peri-ROI | Returned to baseline within 1 min and remained stable | Increased slowly throughout testing | Minor trajectory difference |

**3. Correlation Between Temperature and Erythema Changes**

| **Analysis** | **Published** | **Corrected** |
| --- | --- | --- |
| Overall correlation | r = 0.338, p < 0.001, 95% CI [0.221, 0.445] | r = 0.354, p < 0.001, 95% CI [0.239, 0.459] |
| Intermediate High skin tone | r = −0.421, p = 0.057 (trend) | r = −0.466, p = 0.033* |

**Now statistically significant, strengthening the interpretation that cupping effects may differ by skin tone.*

**4. Effects of Sex, BMI, and Skin Tone on ROI Center Temperature**

No significant differences were found for Sex, BMI Category, or skin tone. 

Figure 4. Sex (a) and BMI category (b) were not associated with significant differences in temperature change following cupping, but trends immediately following cupping were evident. (Revised to use intended 125%/25% axes)

**Summary**

The corrected analysis using 25% axes ROIs yields findings that are consistent with the published manuscript and strengthen several key observations. All regions still show distinct temperature responses, ROI Center still experiences the greatest cooling immediately after cupping and the slowest recovery, and the weak correlation between erythema and temperature persists. The verification analysis provides stronger statistical evidence for some observations (ROI Edge cooling, skin tone differences in correlation).

**All primary conclusions of the study are supported by both analyses.**

**Methods Notes:**

**Published (50%/150% Axes):** ROI Center and Peri-ROI ellipse axes scaled to 50% and ~150% of original ROI axes respectively.

**Verification (25%/125% Axes):** ROI Center and Peri-ROI ellipse axes scaled to 25% and 125% of original ROI axes respectively.
